# LIVEMOS-G: A High Throughput Gantry Monitoring System with Multi-Source Imaging and Environmental Sensing for Large-Scale Commercial Rabbit Farming

**DOI:** 10.3390/ani15213177

**Published:** 2025-10-31

**Authors:** Yutong Han, Tai Wei, Zhaowang Chen, Hongying Wang, Liangju Wang, Congyan Li, Xiuli Mei, Liangde Kuang, Jianjun Gong

**Affiliations:** 1 Rabbit Raising Research Institute, Sichuan Animal Science Academy, Chengdu 610066, China; meixiuli5618@163.com (X.M.); happyboy5851258@163.com (L.K.); gongj2001@126.com (J.G.); 2 College of Engineering, China Agricultural University, Beijing 100083, China; hanyutong0555@cau.edu.cn (Y.H.); wei1135719163@163.com (T.W.); 15650550856@163.com (Z.C.); hongyingw@cau.edu.cn (H.W.); 3 Animal Genetic Breeding and Reproduction Key Laboratory of Sichuan Province, Chengdu 610066, China

**Keywords:** rabbit farming, smart agriculture, inspection system, gantry structure

## Abstract

**Simple Summary:**

Efficient health monitoring in large-scale rabbit farms is essential to reduce economic losses and improve animal welfare. However, traditional manual inspection methods are labor-intensive, prone to error, and struggle to meet the demands of real-time monitoring in high-density farms. In this study, we present a gantry-based inspection system specifically designed for commercial rabbit farming. The system integrates a three-axis motion structure with imaging and environmental sensing modules, enabling automated inspection along fixed paths above the cages. It can acquire high-resolution images and monitor environmental conditions such as temperature, humidity, and light intensity without disturbing the animals. Field experiments show that the system maintains stable movement and achieves complete data collection. A fusion-based network is trained using the collected images and is able to identify dead rabbits with high accuracy, and the network is then deployed to the system for further application. The results demonstrate that the proposed system offers a reliable and non-intrusive solution for real-time monitoring in high-density rabbit houses. It can support early detection of health issues, reduce labor requirements, and promote more intelligent and sustainable farm management practices.

**Abstract:**

The rising global demand for high-quality animal protein has driven the development of advanced technologies in high-density livestock farming. Rabbits, with their rapid growth, high reproductive efficiency, and excellent feed conversion, play an important role in modern animal agriculture. However, large-scale rabbit farming poses challenges in timely health inspection and environmental monitoring. Traditional manual inspections are labor-intensive, prone-to-error, and inefficient for real-time management. To address these issues, we propose Livestock Environmental Monitoring System–Gantry (LIVEMOS-G), an intelligent gantry-based monitoring system tailored for large-scale rabbit farms. Inspired by plant phenotyping platforms, the system integrates a three-axis motion module with multi-source imaging (RGB, depth, near-infrared, thermal infrared) and an environmental sensing module. It autonomously inspects around the farm, capturing multi-angle, high-resolution images and real-time environmental data without disturbing the rabbits. Key environmental parameters are collected accurately and compared with welfare standards. After training on an original dataset, which contains a total of 2325 sets of images (each set includes RGB, NIR, TIR, and depth image), the system is able to detect dead rabbits using a fusion-based object detection model during inspections. LIVEMOS-G offers a scalable, non-intrusive solution for intelligent livestock inspection, contributing to enhanced biosecurity, animal welfare, and data-driven management in high-density, modern rabbit farms. It also shows the potential to be extended to other species, contributing to the sustainable development of the animal farming industry as a whole.

## 1. Introduction

Livestock farming plays a crucial role in global food production, contributing significantly to food security and providing high-quality animal proteins to ensure a balanced diet. As the world’s population is projected to reach 9.8 billion by 2050, the demand for animal-based products—particularly meat, eggs, and dairy—continues to rise [1]. Among various livestock species, rabbits have attracted growing attention due to their fast-growing speed, excellent reproductive capacity, and efficient feed conversion [2]. In turn, this highly productive industry also shows increasing demand for precise and effective farm management.

In traditional livestock farming, animals are inspected and monitored by human workers. Breeders must manually observe the health of animals solely on the basis of their breeding experience to determine the well-being of the rabbits. This has always been a labor-intensive, time-consuming, yet prone to error task, not to mention the risk of disease spread and economic losses due to delayed detection. In the rabbit farming sector, tasks concerning environmental control and death management can be especially challenging due to the specie’s high demanding needs. One key environmental factor is temperature, since rabbits can experience both cold stress and heat stress (heat stress in particular, as rabbits are fur animals). In addition to stress, life threatening diseases occur often in high-density rabbit farms, posing severe challenges to cage-free farm settings, while leveraging the necessity in cage-raising designs where disease control is easier to realize [2]. However, despite the modernization of rabbit rearing practices, housing conditions and other improved farm and animal management practices, mortality rates remain high and disease outbreaks affecting numerical productivity persist [3]. Therefore, with the growth of animal farming industry, manual methods have become increasingly impractical. To address these limitations, there is a growing interest in developing intelligent inspection systems with cutting-edge technologies to support more efficient and reliable management. In recent years, the modernization of animal agriculture has spurred advancements in automation, with technologies such as robotics, precise sensing, and artificial intelligence driving transformative changes in breeding management [4,5]. These developments have given rise to the concept of Precision Livestock Farming (PLF), which aims to manage individual animals through the continuous real-time monitoring of the health, welfare, production, reproduction, and environmental impact [6]. To achieve sustainability and provide better welfare for animals, PLF is especially essential in the commercial livestock farming sector [6,7,8]. Following the trend of PLF, the monitoring systems for farming management have become more intelligent and automated. Upon attaching imaging modules and environmental sensors to mobile inspection systems, the living status and environmental conditions within the farm can be real-time monitored, ensuring both the effective production and the well-being of the animals. By further applying image processing techniques, breeders are able to observe the living status, behaviors, as well as health conditions of animals without having to frequently go through the houses. As the collected information is objectively precise, these systems offer more reliable and autonomous alternatives, boosting the overall efficiency and productivity.

In high-density farming environments, intelligent monitoring systems are developed to address the challenges of efficient and accurate inspections. Fixed systems, such as ceiling mounted surveillance cameras, are widely used due to their simple installation and low cost [9,10]. For example, Zhu et al. [11] fixed cameras in front of chicken cages to capture images at five-minute intervals. By comparing sequential image pairs, they extracted movement-related features for behavior analysis, with the fixed setup ensuring that variations were caused by the animals rather than external factors. Xu et al. [12] acquired images and videos from surveillance cameras to identify aggressive behavior in group sheep. The fish-eye camera they chose provided a comprehensive view within the sheep pen. Bist et al. [13] installed eight night-vision network cameras to monitor cage-free hen mortality, with six cameras mounted 3 m above the litter floor, one at 0.5 m, and another at 1 m ground heights. However, the setups of such systems often suffer from limited coverage, occlusion by animals or cage structures, and less-satisfying image resolution when observing distant targets, making them less reliable for detailed inspections.

To overcome these limitations, mobile inspection platforms have been introduced, offering improved adaptability and closer-range data acquisition. Equipped with advanced navigation technologies like LiDAR and SLAM, these mobile systems can autonomously navigate around farm houses while capturing high-resolution images [13,14,15,16,17,18]. For instance, Ma et al. [15] developed a mobile device with integrated QR-code-based positioning to navigate accurately through poultry farms. This robot achieves both precise localization and efficient data acquisition, significantly reducing labor while maintaining high detection accuracy (90.61%) in dead chicken detection tasks. Yang et al. [14] collected images of both dead and alive chickens with an inspection robot. The trained dead chicken detection algorithm was then deployed to the device, and proved to achieve high detection accuracy at an inspection speed of 0.2 m/s. However, as most of the rabbit houses adopt aisles less than 0.8 m in width, it can be quite challenging for general inspection robots to pass, not to mention performing inspection tasks. Moreover, as the imaging sensors of the mobile device is generally installed on a fixed height, limitations in the diversity of viewpoints can result in reduced coverage of both upper and lower cage levels.

In facing these existing limitations in predominant systems, gantry-based inspection systems offer a promising alternative. Inspired by greenhouse-based phenotyping platforms in plant research, gantry systems present a reliable solution for structured and stable data collection in animal farming. In plant phenotyping applications, gantry systems are widely used to support sensor-to-target imaging in greenhouse or field environments, offering benefits such as fixed path operation, minimal interference with the biological subjects, and high-precision, as well as repeatable imaging from customizable viewpoints. These platforms typically integrate multi-modal sensors, including RGB, depth, and thermal cameras, to acquire high quality datasets for phenotyping analysis. For example, Zhou et al. [19] developed a gantry-type phenotyping platform for greenhouse environments, featuring a ceiling-mounted XY sliding track system, stepper motor-driven motion modules, and a sensor mount for image acquisition. The sensor unit moves in two perpendicular directions along aluminum tracks, allowing programmable, repeatable scanning over crops. Vibration-damping components and ball-bearing sliders ensured smooth and stable movement. The system achieved full-area coverage with high image overlap by precisely controlling movement speed and positioning, while the ceiling-mounted design allowed easy integration into existing greenhouse structures. Du et al. [20] designed a greenhouse-based high-throughput phenotyping platform with a four-degree-of-freedom imaging unit (X, Y, Z, and rotation), capable of precise and automated image acquisition. The system was mounted on load-bearing columns of a conventional terraced greenhouse and featured a large-span XY dual-track beam structure for overhead navigation. A railcar equipped with a five-stage telescopic lifting mechanism and a 360° rotatable imaging box enabled dynamic adjustment of imaging height (1.4–3.8 m) and angle. The platform supported programmable movement along predefined routes and operated under an integrated control architecture using industrial PLCs and MODBUS TCP/IP communication. With this configuration, the system enabled continuous, high-resolution top-view imaging of over 2000 lettuce plants, offering accurate spatial localization and consistent image quality across growth stages. The NU-Spidercam platform, developed by Bai et al. [21] is a cable-driven field phenotyping system that enables automated 3D movement of a sensor unit over a 0.4 ha crop area with ±5 cm accuracy. Suspended by eight Kevlar cables from four 27 m towers, the platform supports multi-modal imaging (RGB-NIR, thermal, LiDAR, spectrometer) and operates at heights from 0 to 9 m. A built-in positioning algorithm allows precise navigation without GPS. The system enables rapid, high-throughput data collection over tall crops, supporting advanced field phenotyping under real conditions. Adapting the gantry concept into high-density livestock farming, particularly in caged environments such as rabbit or poultry houses, provides new opportunities to overcome the challenges of occlusion and inconsistent imaging angles. By operating above the animal cages and traveling along a predefined inspection path, gantry-based monitoring systems can ensure full coverage of the farm with minimal disturbance to the animals. Moreover, the designed routes and height-adjustable sensor modules make it possible to achieve multi-view and close-range imaging, which is critical for tasks such as death detection and animal behavioral analysis.

In this study, we present LIVEMOS-G, a gantry-type inspection system specifically developed for large-scale rabbit farming. The system integrates a three-axis motion structure (X, Y, Z), a multi-source imaging module (RGB, depth, near-infrared, and thermal infrared), as well as an environmental sensing module. It is capable of autonomously inspect throughout the rabbit house, collecting imaging and environmental data from multiple perspectives, and further supporting real-time data transmission for on-site analysis. The mechanical design has been recognized for its great stability and adaptability to complex farm structures, while the imaging unit is capable of supporting high-resolution, multi-angle observations of both upper and lower cages. Details regarding the specific hardware designs and its control system are presented in Section 2. On-site experiments are conducted to validate the system’s inspection performance, motion reliability, and application potential in real farming scenarios.

Existing fixed monitoring systems and ground-based mobile robots have shown notable progress in livestock inspection, yet both approaches face challenges when applied to high-density rabbit farming. Fixed systems often suffer from occlusion by cage structures, limited field of view, and insufficient image resolution for detailed observation. Meanwhile, mobile inspection robots, although capable of autonomous navigation, are constrained by the narrow aisles of rabbit houses and the fixed height of their imaging modules, which restricts multi-level inspection and viewpoint flexibility. To address these challenges, the development of LIVEMOS-G fills a critical gap in precision livestock management by integrating the structural advantages of a gantry-based platform with multi-source imaging and precise environmental sensing. This system enables stable, close-range, and multi-angle inspections across dense cage environments, providing a scalable and intelligent solution tailored to the operational demands of modern rabbit farming.

## 2. Materials and Methods

### 2.1. Experimental Base

**Figure 1:**
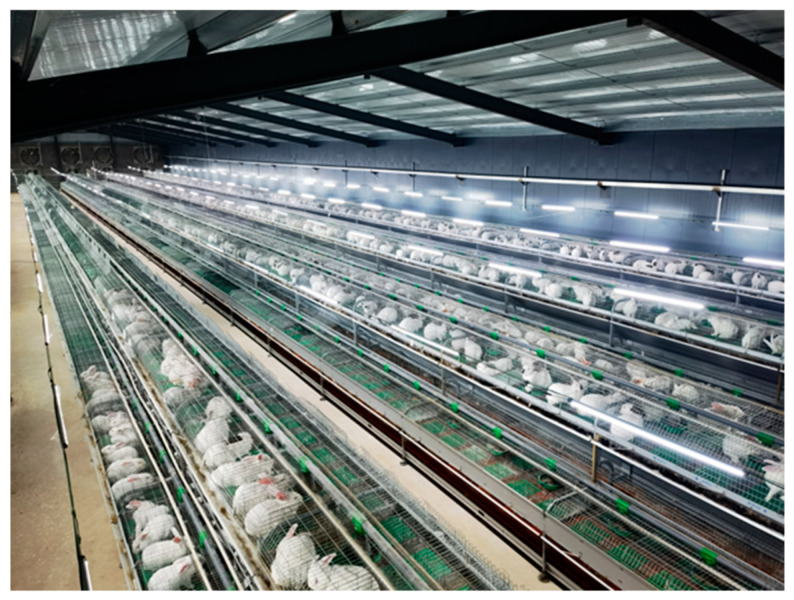
Experimental environments in which the developed system was installed.

The gantry monitoring system is installed and tested in a commercial rabbit farm operated by Shandong Tutu Breeding Co., Ltd., Linyi, Shandong Province, China. As shown in Figure 1, the rabbit house measures approximately 72 m in length and 13.5 m in width, featuring a sloped roof design. The lowest point of the roof is three meters above the ground, while the eaves reached 3.6 m. There are four rows of double-sided cages arranged with a one-meter spacing between rows. Each row consists of two stacked layers, forming a pyramid-like structure.

### 2.2. Design of the Gantry System

The LIVEMOS-G monitoring system consists of three main components, the XYZ-axis motion module, the multi-source imaging module, and the environmental sensing module. In this section (Section 2.2), the specific designs of each module and how the system works in real-world scenarios are presented with details.

The gantry inspection system operates within a workspace measuring 58 m (length) × 13.3 m (width) × 0.8 m (height) and features a three-degree-of-freedom (DOF) X-Y-Z gantry motion structure. It supports fully automated, continuous 24 h operation, supporting inspection tasks of both environmental and livestock conditions. The system achieves a positioning accuracy of ±10 mm, with adjustable travel speeds ranging from 0.05 to 0.3 m/s along the X-axis, and a constant speed of 0.05 m/s along the Y- and Z-axes. To ensure stable and synchronized motion of the dual-side X-axis drive units under real-world conditions, an automatic deviation correction system based on encoder feedback and closed-loop control is implemented, allowing real-time compensation for rail unevenness, wheel diameter differences, and asymmetric loads, thereby ensuring safe and stable operation throughout the inspection process. The data acquisition module includes multi-modal imaging sensors capturing RGB, thermal infrared, near-infrared, and depth images, complemented by environmental sensors measuring CO_2_, NH_3_, temperature, illumination intensity, atmospheric pressure, and wind velocity.

#### 2.2.1. XYZ-Axis Motion Module

**Figure 2:**
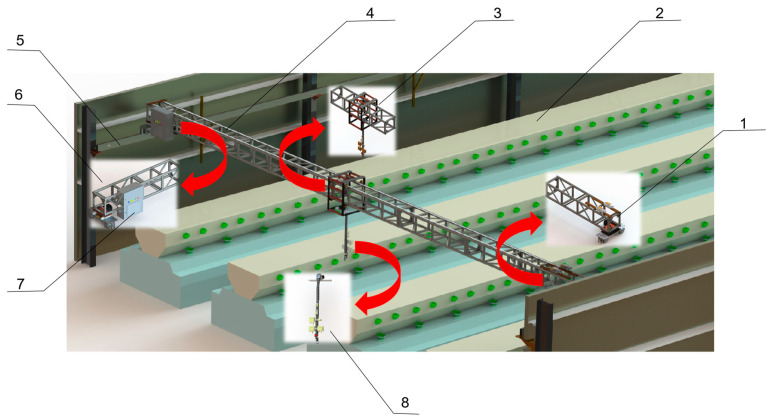
The overall hardware design of LIVEMOS-G system. (1) Adjustable motion unit of X-axis motion module; (2) Rabbit cages; (3) Y-axis motion module; (4) Y-axis beam; (5) Fixed motion unit of X-axis motion module; (6) Fixed motion unit of X-axis motion module; (7) Electrical control cabinet; (8) Z-axis multi-sensing module.

The LIVEMOS-G system comprises a three-axis (XYZ) motion module for precise axis-specific motion control, as shown in Figure 2. Each axis-specific module integrates with sensors and actuators to ensure the overall stability and accuracy of the hardware system.

**Figure 3:**
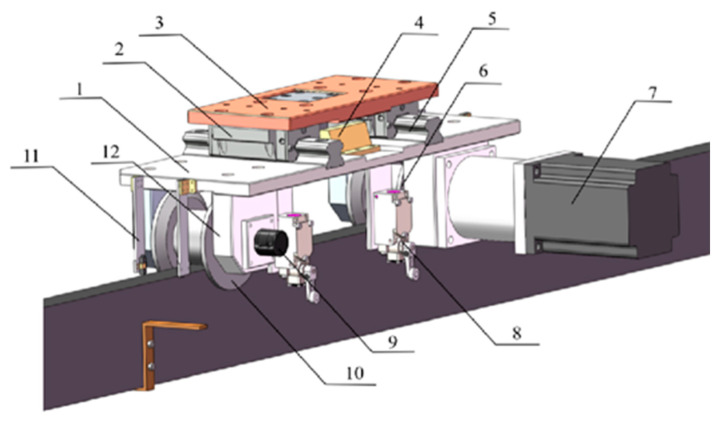
Hardware design of the adjustable motion unit. (1) Main board; (2) Slider; (3) Y-axis beam connector; (4) Adjustable motion stopper; (5) Linear guide; (6) Active wheel; (7) X-axis left motor; (8) Limit switch; (9) Left encoder; (10) Passive wheel; (11) Gap sensor; (12) Wheel support.

**Figure 4:**
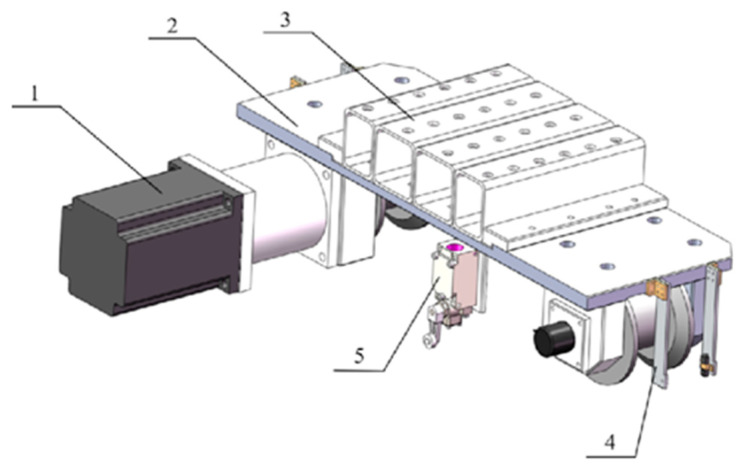
Hardware design of the fixed motion unit. (1) X-axis right motor; (2) Main board; (3) Steel square tube spacer; (4) Gap sensors; (5) Limit switch.

Forming the foundation of the gantry, the X-axis module of the LIVEMOS-G system is composed of two side rails and a set of motion modules. The side rails are 50 m in length, stretching across the rabbit house in X-axis direction, and the motion module is designed to provide stable, precise movement along these rails. There are two motion units in total, with one attached to each rail, forming the motion module. The adjustable motion unit, shown in Figure 3, adopts a distance adjustment mechanism, designed to adapt to the variations in rail spacing within a specific range. Whereas the fixed motion unit, shown in Figure 4, which is attached to the other rail, is rather fixed to the beam, ensuring the stability of the system. Each motion unit includes travel limit switches beneath its main board to enhance positioning accuracy. The adjustable unit includes two such switches, mounted at the front and rear of the board, which are triggered upon contact with limit stoppers at the start and end of the X-axis rail, thereby generating travel limit signals. In contrast, the fixed unit is equipped with a single switch that activates upon reaching the origin stopper, generating an origin limit signal. Power transmission for the X-axis is achieved through a drive motor and a reducer, which are connected to the active wheel, while a absolute encoder is fixed to the passive wheel, tracking the distance information along the X-axis. A total of four gap sensors is equipped to the motion modules, one on each wheel, sensing the distance between the wheels and the rail to ensure its stability.

**Figure 5:**
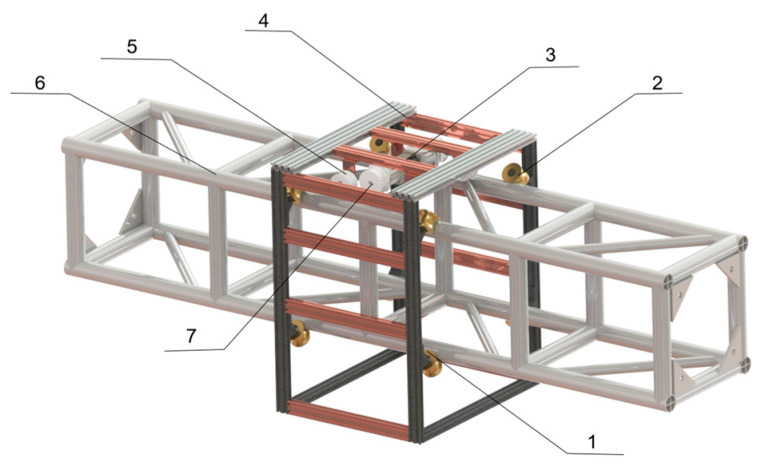
Hardware design of the Y-axis motion module. (1) U-shaped wheel; (2) U-shaped wheel support; (3) Driving module; (4) Aluminum frame; (5) Tensioner pulley; (6) Y-axis beam; (7) Timing belt pulley.

As shown in Figure 5, the Y-axis module includes a 13 m long aluminum alloy beam, extending across the rabbit house’s width, and the Y-axis motion module. The beam stretches across the two parallel rails in Y-axis direction by fixing the ends to the motion units moving along these two separate rails (the motion units of the X-axis motion module). It is constructed from aluminum truss structures with a 400 × 400 mm cross-section, providing both structural stability and lightweight characteristics. The Y-axis motion module is symmetrically designed along the central axis of the beam to ensure balanced operation. The module structure is slightly larger than the beam. Therefore, by using eight recessed U-shaped rollers to interface with the circular tubes of the beam at a 45° contact angle, the resultant force can be directed towards the center for optimal alignment. Among these eight rollers, four upper ones bear the vertical resultant load, while the four lower ones are designed to prevent tilting or derailing during accidental collisions. The Y-axis motion module is driven by a dual-shaft motor system, delivering synchronized and smooth movement along the Y-axis.

**Figure 6:**
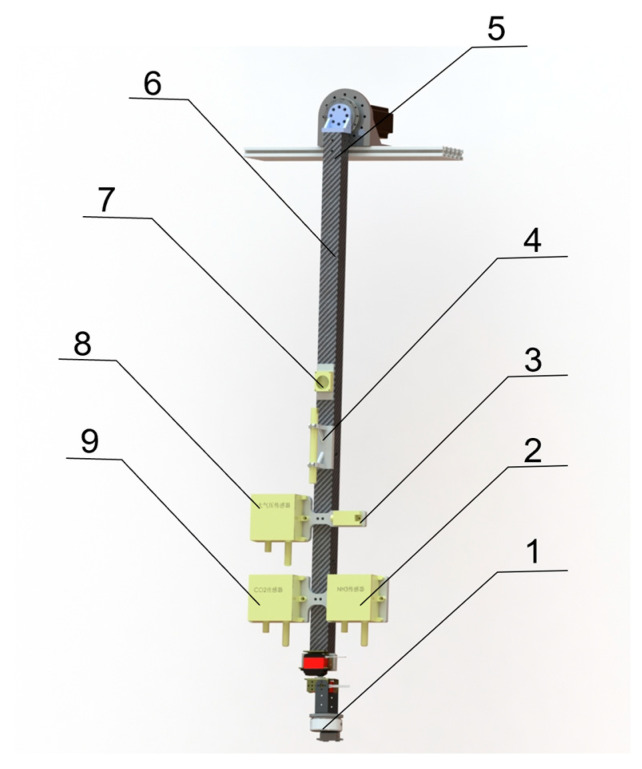
Hardware design of the Z-axis module. (1) Multi-source imaging module; (2) NH_3_ sensor; (3) Temperature humidity sensor; (4) Wind speed sensor; (5) Z-axis motion module; (6) Carbon Fiber rod; (7) Light sensor; (8) Air pressure sensor; (9) CO_2_ sensor.

The Z-axis motion module is mounted on the lower end of the Y-axis motion module. As presented in Figure 6, the Z-axis module consists of a motion unit, an imaging module, and an environmental sensing module. The motion unit relies on an 800 mm single-joint arm to rotate within the Y-Z plane. This movement enables variations in imaging angles, as well as image acquisition of multiple cage rows within the same aisle by simply adjusting the orientation of the arm. The design also ensures that the Z-axis module, as well as the whole gantry system, can bypass obstacles such as overhead lighting structures, achieving smooth and consistent inspection tasks. The imaging module and the environmental sensing module are presented with details in the following sections (Section 2.2.2 and Section 2.2.3). The entire structure is centrally installed at the bottom of the Y-axis motion module to maintain balance and stability of the system.

#### 2.2.2. Multi-Source Imaging Module

**Figure 7:**
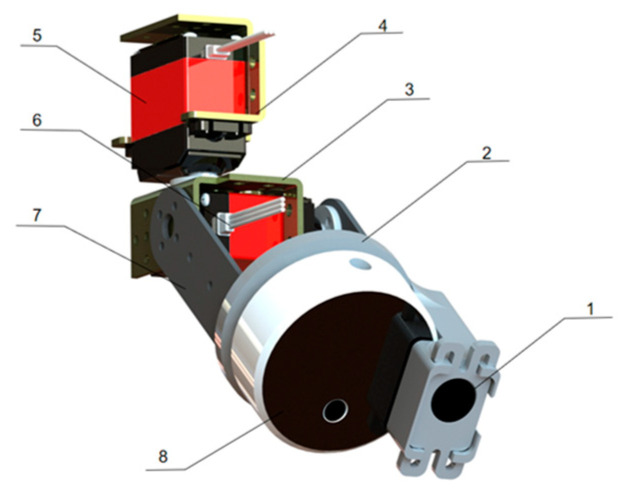
Hardware design of the multi-source imaging module. (1) Thermal camera; (2) Cameras connector; (3) Lower servo support; (4) Upper servo support; (5) Upper servo; (6) Lower servo; (7) Camera support; (8) Depth camera.

As shown in Figure 7, the multi-source imaging module of LIVEMOS-G consists of a two-degree-of-freedom (2-DoF) pan-tilt unit, a light detection and ranging (LiDAR) camera (RealSense L515, Intel Corporation, Santa Clara, CA, USA), and a thermal infrared camera (IRay P2, IRay Technology Co., Ltd., Yantai, China). The 2-DoF pan-tilt unit is composed of two servo motors, one controlling pitch (vertical rotation) and the other controlling yaw (horizontal rotation), ensuring the comprehensive image acquisition of the stacked rabbit cages.

#### 2.2.3. Environmental Sensing Module

The environmental sensing module of LIVEMOS-G is integrated with six precise sensors, including a carbon dioxide sensor (HSTL-CO2, Beijing HuaKong Xingye Technology Development Co., Ltd., Beijing, China), and ammonia sensor (HSTL-NH3, Beijing HuaKong Xingye Technology Development Co., Ltd., Beijing, China), a wind speed sensor (W410C2, Hangzhou Jiahan Electronics Co., Ltd., Hangzhou, China), a temperature and humidity sensor (SHT30, Sensirion, Stäfa, Switzerland), an atmospheric pressure sensor (BITS105), and a light intensity sensor (TSL25911, ams-OSRAM AG, Premstaetten, Austria). These sensors are responsible for monitoring key environmental parameters inside the rabbit house. Details regarding these sensors are presented in Table 1.

### 2.3. Workflow

#### 2.3.1. Hardware Architecture

The LIVEMOS-G system employs an Intel Next Unit of Computing (NUC, Intel Corporation, Santa Clara, CA, USA) as the main control, while a Siemens SIMATIC S7-200 SMART CPU ST30 programmable logic controller (Siemens AG, Munich, Germany) functions as the secondary controller. The ESP32-S3-WROOM-1 module (Espressif Systems, Shanghai, China) serves as the environmental data acquisition controller.

**Figure 8:**
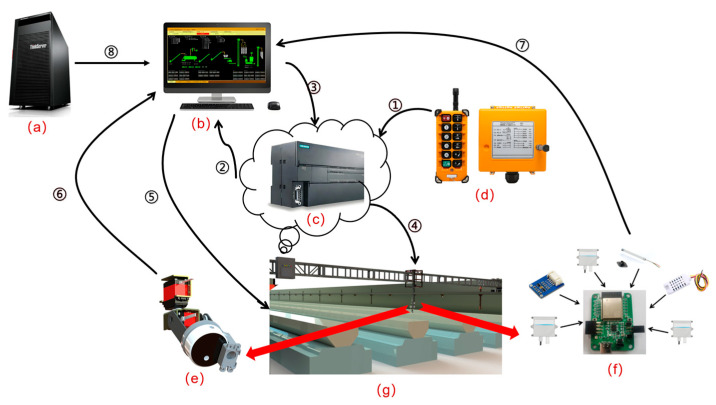
Hardware architecture of the LIVEMOS-G system. (**a**) server; (**b**) computer; (**c**) PLC; (**d**) industrial controller; (**e**) imaging module; (**f**) environmental sensing module; (**g**) gantry system. The overall hardware architecture is presented and its workflow with arrows ① to ⑦. ① the action of pressing a specific button on the industrial controller to update a register value in the PLC; ② the real-time monitoring of this register by upper computer. ③ when a change is detected, the upper computer writes a transient switching signal to another PLC register. ④ upon receiving this signal, the PLC activates the gantry system and controls the sensing modules to move to the designated coordinate. ⑤ upon reaching the target position, the upper computer commands the multi-source imaging and environmental sensing modules to start data acquisition. ⑥ ⑦ the simultaneous capture of image and environmental data, which are then uploaded to a specific storge directory on the upper computer for subsequent data processing tasks. ⑧ the upper computer connects to the server via Ethernet, while the server functions as a database and processes images.

For data communication, the ESP32 module supported the ICC (Inter-Integrated Circuit) protocol to interface with temperature-humidity sensors and illuminance sensors. It also supported the MODBUS protocol to exchange data with the wind speed, barometric pressure, ammonia, and carbon dioxide sensors. The overall hardware architecture of the LIVEMOS-G system is presented in Figure 8.

#### 2.3.2. Software Structure

The control system of the LIVEMOS-G is designed to achieve a full-time, fully automated inspection around the rabbit house. Building on the aforementioned hardware architecture, an automatic inspection program is implemented on the host computer to achieve both accurate motion control and comprehensive multi-source data acquisition. This program integrates Python-based (Python 3.10.15) motion control routines with dedicated modules for imaging and environmental sensing.

**Figure 9:**
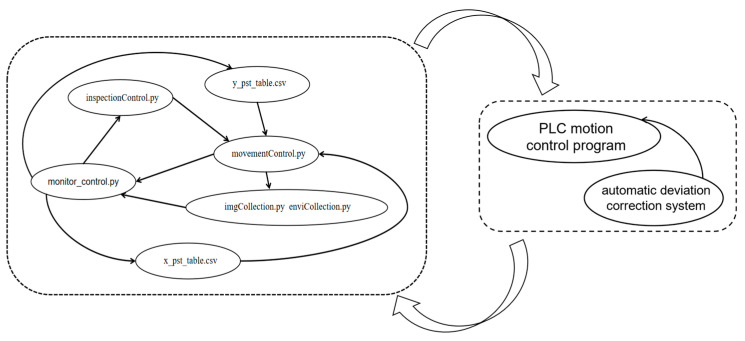
Software structure of the LIVEMOS-G system.

Within the automative inspection control program shown in Figure 9, the core control module (monitor_control.py) serves as the main program, responsible for coordinating the overall task flow. The inspection module (inspectionConrtol.py) executes the inspection movement commands received from the core module. The motion control module (movementControl.py) carries out the mechanical operations based on the commands from the inspection module and the predefined route coordinate tables (x_pst_table.csv and y_pst_table.csv). Meanwhile, the multi-source imaging module (imgCollection) and the environmental data collection module (enviCollection.py) acquire visual and environmental data, respectively, and transmit these data back to the core control module. All modules communicate via standardized interface communications to ensure data consistency and real-time performance.

**Figure 10:**
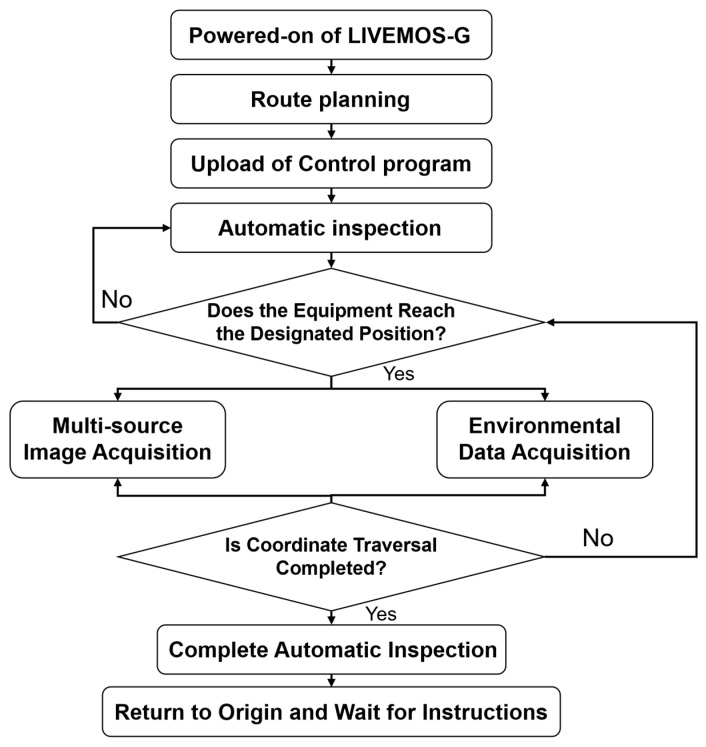
Overall workflow of the inspection process.

Once LIVEMOS-G is powered-on at its starting position, it waits for inspection commands. Upon receiving the signal, the system follows the predesigned inspection route. At each set position, the system halts its movement and performs multi-source data collection, including the acquisition of multi-source images and environmental data. After completing data collection at the current position, the system proceeds to the next, repeating the same procedure until the entire route is finished. Finally, the inspection unit returns to the starting position, completing the full inspection process. The overall workflow is illustrated in Figure 10.

Based on the control procedure shown in Figure 10, the whole process can be divided into three primary tasks, motion control, multi-source image acquisition, and environmental data collection. In this section (Section 2.3.2), details regarding these processes will be presented.

(1). Motion control module

**Figure 11:**
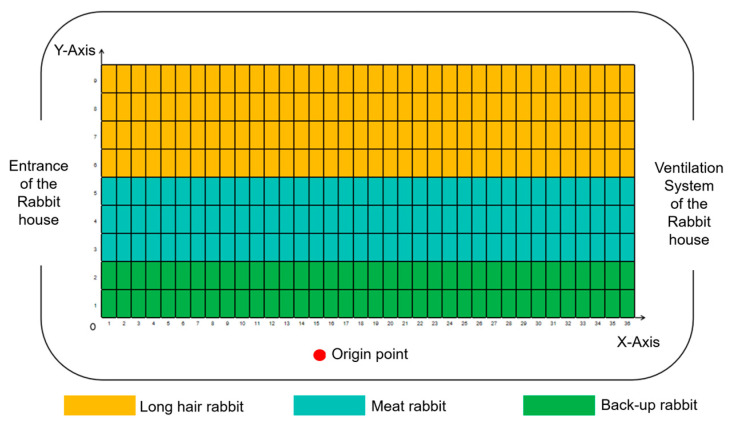
Predefined coordinate system of the rabbit house.

Based on a predefined coordinate system of the rabbit house shown in Figure 11, the inspection route of the LIVEMOS-G system is set in advance using the monitor_control.py program.

The host computer communicates with the PLC controller. Upon receiving the inspection command, the host initiates a homing operation by writing specific values to designated PLC registers. The inspection device then begins executing the homing procedure. Once the device triggers the limit switch, the homing process completes. The host computer then proceeds to launch the inspection routine, sending absolute pulse values to the PLC to move the device to the first target location.

**Figure 12:**
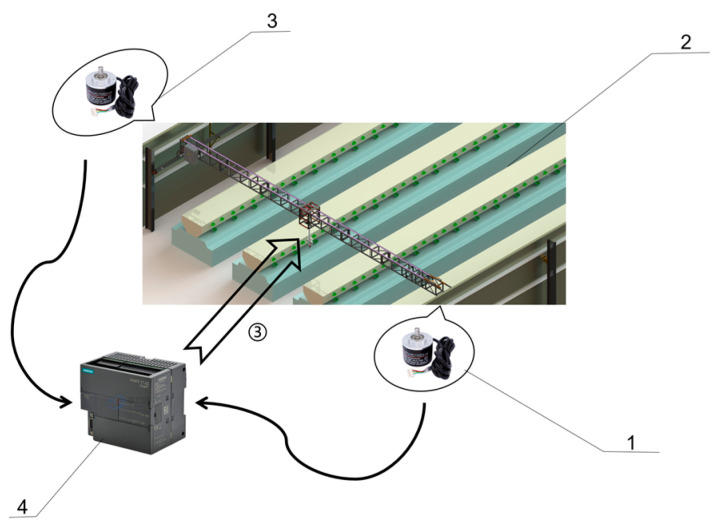
The control system of automatic deviation correction system. (1) Right-side absolute encoder; (2) Inspection system; (3) Left-side absolute encoder; (4) PLC controller.

During inspection, various factors may affect the working consistency of LIVEMOS-G, such as uneven rails, wheel diameter differences, speed differences in motor and reducer, as well as asymmetric load distribution. These factors may cause desynchronization between the adjustable motion unit and fixed motion unit (both components of the X-axis motion module) on the two sides of the gantry. Excessive deviation can lead to rail misalignment or, in extreme cases, equipment tipping, posing serious safety risks. Therefore, when deviations exceed a defined threshold, automatic correction is required to maintain safe and stable operation. To address this, a closed-loop control system is established using feedback from absolute encoders on both motion units shown in Figure 12. 

The adjustable motion unit on the left X-axis rail is set as the reference while fixed motion unit is constantly under adjustments accordingly. Programs are applied to control the forward and backward movement. The system monitors the relative deviation by continuously reading encoder feedback signals. Encoder values from both sides are sent to PLC registers VD300 and VD500. The PLC then calculates the absolute difference between the two values and compares it to predefined thresholds to assess the status of the system.

When the deviation is less than 50 mm, the system is believed to be in proper function and continues the inspection task. When the deviation ranges between 50 mm and 70 mm, slight misalignment is detected. The system determines the direction of motion and which side leads. It then adjusts the speed of the fixed unit on the right to either 120% or 80% of its current speed based on the detected status. When the deviation is between 70 mm and 100 mm, mass misalignment appears. To address this, the system increases or decreases the speed of the fixed motion unit to 140% or 60% of its current speed, making up for the condition. If the deviation exceeds 100 mm, the system stops immediately and triggers an alarm, calling for human intervention and correction. This automated deviation correction mechanism enables real-time monitoring and adjustment of motion synchronization, ensuring safe and stable operation throughout the inspection task.

Once the XYZ axis reaches the designated position, the device stops, and the host computer sends commands to trigger multi-source image acquisition and environmental sensing. After data collection at the current location is completed, the host writes the next position command to the PLC, and the inspection device continues moving along the predesigned route. When the entire preplanned path is completed, the device halts and awaits the next inspection instruction.

(2). Multi-source image acquisition

The multi-source imaging module includes a depth camera (RealSense L515, Intel Corporation, Santa Clara, CA, USA) and a thermal infrared (TIR) camera (IRay P2, IRay Technology Co., Ltd., Yantai, China), supporting the simultaneous collection of four types of visual data, including near-infrared (NIR) images (1024 × 768 pixels, 30 fps), RGB images (1920 × 1080 pixels, 30 fps), depth images (1024 × 768 pixels, 30 fps), and TIR images (256 × 192 pixels). Both cameras connect to the host computer via USB interfaces, with image data automatically transmitted and stored in designated directories. During the experiment, the rabbit housing area is mapped onto an X-Y coordinate comprising 78 points along the X-axis and 12 along the Y-axis. Each coordinate point corresponds to a group of six cages. Captured images are named following a standardized format, namely, imageType_date_time_x_y. For example, a depth image taken at coordinate (5,6) is labeled depth_20240729_1228_5_6, encoding the image type, acquisition date, timestamp, and spatial location.

The inspection system stops upon reaching a target position. The host computer then retrieves real-time coordinate values from PLC registers to determine its current position along the X and Y axis. This information is then used to identify the specific row, column, and tier of the target cage. The system subsequently adjusts the height and orientation of the imaging module to align with the target cage area.

**Figure 13:**
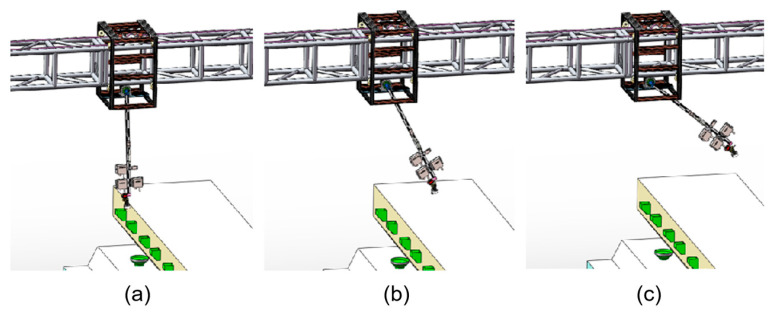
Possible operating postures of the Z-axis module. (**a**) Collecting data of lower cages; (**b**) Collecting data of upper cages; (**c**) passing power wire of light.

During the inspection, the Z-axis module supports three operating postures, as shown in Figure 13.

**Figure 14:**
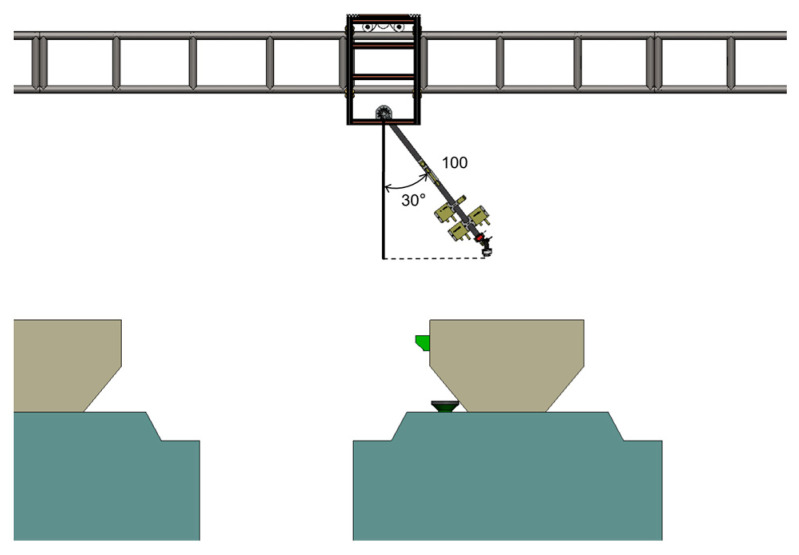
Possible operating postures of the Z-axis module when acquiring images of the upper coop.

For lower-tier data collection, the Z-axis remains vertical to the ground, and thus no control pulses or digital triggers are sent. Whereas when imaging upper cages, the Z-axis arm rotates to an angle of 30°, shown in Figure 14, extending the effective arm length to approximately 86.6 cm. This relative position ensures that the camera is approximately 38.4 cm above the cage, satisfying the imaging distance requirements.

**Figure 15:**
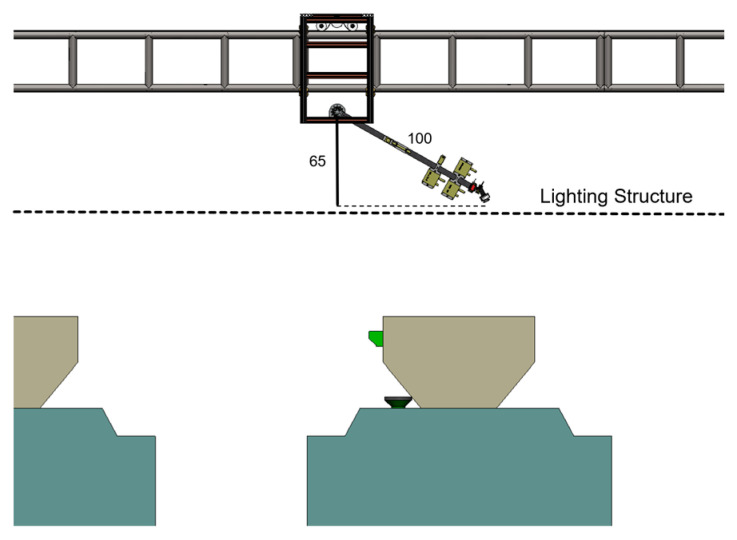
Possible operating postures of the Z-axis module when acquiring images of the lower coop.

As illustrated in Figure 15, the Z-axis module is also capable of bypassing the lighting structures of the rabbit house. When lifting the Z-axis module to a horizontally 65 cm from the Y-axis module, the system is able to avoid obstructions, ensuring smooth movement and consistent data acquisition.

(3). Fusion-based dead rabbit detection using near-infrared and thermal imaging

In this study, we trained a fusion-based dead rabbit detection model under the operating scenarios of the LIVEMOS-G system and further deployed the model for on-site experiments. In commercial rabbit houses, light intensities are typically quite low. Under such conditions, NIR images can effectively capture the contours and locations of rabbits without relying on supplementary lighting, whereas TIR images provide temperature information that serves as a reliable indicator of death. By fusing these modalities into multi-source datasets, detection accuracy can be enhanced through the complementary strengths of these image types. Because the TIR camera had a smaller field than the NIR camera, in order to retain more information after registration, both NIR and TIR image coordinates are projected onto the TIR image coordinate system. Given the fixed relative positions between the cameras, the registration process followed a standardized multi-step coordinate transformation pipeline, incorporating both intrinsic and extrinsic parameters obtained under a unified world coordinate system.

To achieve pixel-level registration between the NIR and TIR images, the three-step coordinate transformation is as follows.

(a) NIR image to NIR camera coordinates.

NIR intrinsic parameters ${\left[k_{x},k_{y},u_{0},v_{0}\right]}_{\mathrm{NIR}}$ and depth information $Z_{\mathrm{NIR}}$ convert NIR pixel coordinates ${\left[u,v\right]}_{\mathrm{NIR}}$ into 3D coordinates ${\left[X,Y,Z\right]}_{\mathrm{NIR}}$ in the NIR camera coordinate system. The camera coordinate system coordinates from image coordinates could then be calculated with the assistance of depth information the imaging module contains:

(1) $$X_{\mathrm{NIR}}=\frac{\left(u_{\mathrm{NIR}}-u_{0}^{\mathrm{NIR}}\right)\cdot Z_{\mathrm{NIR}}}{k_{x}}\;Y_{\mathrm{NIR}}=\frac{\left(v_{\mathrm{NIR}}-v_{0}^{\mathrm{NIR}}\right)\cdot Z_{\mathrm{NIR}}}{k_{y}}$$

where *Z*_NIR_ is obtained from the aligned depth image.

(b) NIR to TIR camera coordinates.

Equations (2) and (3), as shown below, are used to transform parameters in the NIR camera coordinate system and the TIR camera coordinate system to the world coordinate system.

(2) $${\left[\begin{array}{c} X\\ Y\\ Z \end{array}\right]}_{\mathrm{NIR}}=R_{\mathrm{NIR}}{\left[\begin{array}{c} X\\ Y\\ Z \end{array}\right]}_{W}+p_{\mathrm{NIR}}$$

(3) $${\left[\begin{array}{c} X\\ Y\\ Z \end{array}\right]}_{\mathrm{TIR}}=R_{\mathrm{TIR}}{\left[\begin{array}{c} X\\ Y\\ Z \end{array}\right]}_{W}+p_{\mathrm{TIR}}$$

where ${\left[X,Y,Z\right]}_{\mathrm{NIR}}$ is the coordinate in the NIR camera coordinate system, ${\left[X,Y,Z\right]}_{W}$ is the coordinate in the world coordinate system, ${\left[X,Y,Z\right]}_{\mathrm{TIR}}$ is the coordinate in the TIR camera coordinate system, R_NIR_ is the rotation matrix of the NIR camera, R_TIR_ is the rotation matrix of the TIR camera, P_NIR_ is the translation vector of the NIR camera, and P_TIR_ is the translation vector of the TIR camera.

As the images are captured in the same world coordinate system, the ${\left[X,Y,Z\right]}_{W}$ in the equations are the same. Therefore, based on Equations (2) and (3), Equation (4) can be derived.

(4) $${\left[\begin{array}{c} X\\ Y\\ Z \end{array}\right]}_{\mathrm{TIR}}=R_{\mathrm{TIR}}\left(R_{\mathrm{NIR}}^{-1}\left({\left[\begin{array}{c} X\\ Y\\ Z \end{array}\right]}_{\mathrm{NIR}}-p_{\mathrm{NIR}}\right)\right)+p_{TIR}$$

This equation transforms coordinates in the NIR camera coordinate system into the coordinates in the TIR camera coordinate system.

(c) TIR camera coordinates to TIR image.

Similar to (a), but conversely, coordinates in the TIR camera coordinate system are transformed into the TIR image coordinates using Equation (5).

(5) $${\left[\begin{array}{c} u\\ v\\ 1 \end{array}\right]}_{\mathrm{TIR}}={\left[\begin{array}{ccc} k_{X} & 0 & u_{0}\\ 0 & k_{y} & \nu_{0}\\ 0 & 0 & 1 \end{array}\right]}_{\mathrm{TIR}}{\left[\begin{array}{c} X/Z\\ Y/Z\\ 1 \end{array}\right]}_{\mathrm{TIR}}$$

where ${\left[u,v\right]}_{\mathrm{TIR}}$ is the TIR image coordinates, ${\left[k_{x},k_{y},u_{0},v_{0}\right]}_{\mathrm{TIR}}$ is the intrinsic parameters of the TIR camera, and ${\left[X,Y,Z\right]}_{\mathrm{TIR}}$ is the coordinate in the TIR camera coordinate system.

Therefore, through steps (a) to (c), the transformation from the NIR image coordinates to the TIR image coordinates can be achieved. The registration process described above is implemented using Python and the OpenCV-Python library.

For object detection, the YOLO (You Only Look Once) framework offers an efficient solution by unifying bounding box regression and classification into a single neural network, thereby enabling end-to-end training and real-time inference. Recent advances in state-of-the-art computer vision have further strengthened this foundation, with the continuous evolution from YOLO series to transformer-based models such as Vision Transformer (ViT) and multimodal fusion frameworks that integrate RGB, thermal infrared, and depth information for enhanced robustness and adaptability [22,23,24]. These developments demonstrate the rapid progress of deep learning methods toward more generalizable, data-efficient, and interpretable systems suitable for agricultural environments. In the dead animal detection sector of precision farming and smart agriculture, YOLO has been proven to perform well, especially for real-time detection tasks. When performing dead chicken detection tasks with their self-developed inspection robot, Yang et al. [14] proposed an improved YOLOv7 model, presenting a new network structure and loss function. The precision, recall, and mAP@0.5 for the dead hens in the test set achieved 95.7% and 86.8%. Luo et al. performed image registration and fusion with NIR, TIR, and depth image with YOLOv8, their AP reached 98.6% (IoU = 0.5) and recall achieved 97.3% (IoU = 0.5) [16].

Among the recent iterations of the YOLO series, YOLOv11 represents a major advancement, achieving superior performance in both speed and accuracy compared with earlier versions. Its architecture maintains the backbone–neck–head structure but introduces several innovations, including the C3k2 (Cross-Stage Partial Block with kernel size 2), C2PSA (Convolutional block with parallel Spatial Attention), and SPPF (Spatial Pyramid Pooling–Fast) modules [24]. These modules collectively improve feature extraction, enhance spatial attention, and accelerate inference, making YOLOv11 particularly effective in complex detection scenarios. Specifically, the backbone of YOLOv11 employs C3k2 blocks to strengthen multi-scale feature extraction, while the neck integrates C3k2 and C2PSA modules to accelerate feature fusion and reduce computational cost. The detection head further refines feature maps through multiple C3k2 blocks, achieving higher precision with lightweight computation. Together, these enhancements highlight the excellent performance of YOLOv11 in terms of accuracy, speed, and efficiency for real-time detection tasks. Therefore, in this research, YOLOv11 is selected as the baseline of the deployed dead rabbit detection model.

(4). Environmental data collection

The environmental data are automatically acquired at the same time while multi-source images are collected. An environmental controller, based on the ESP32-S3-WROOM-1 module, runs the acquisition program, while corresponding data processing program operates on the host computer.

The environmental sensing program, developed using the Arduino language (Arduino IDE 2.3.2), initializes the microcontroller, collects environmental data, such as temperature, humidity, and applies unit conversions.

On the host computer, the environmental data processing program, implemented in Python 3.10.15, receives data transmitted from the environmental controller, organizes the information for clear structure, and stores them in Comma-Separated Values (CSV) files. Each CSV file contains six types of environmental data, the time at which data are collected, and the CSV files of the cage coordinates.

### 2.4. Experimental Evaluation

#### 2.4.1. Mechanical Stability of the Inspection System

**Figure 16:**
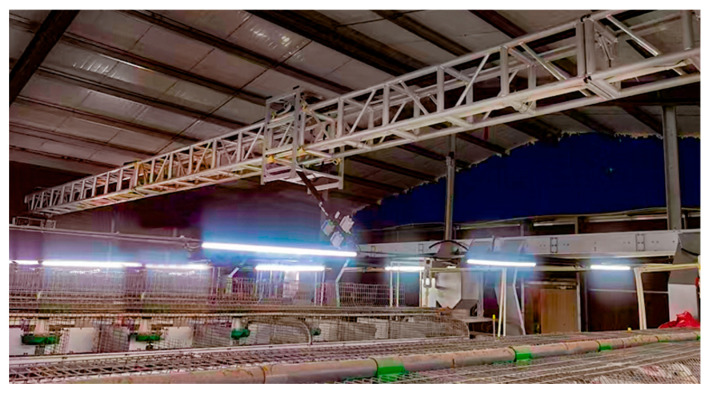
Scene from the on-site experiments.

The mechanical stability of the inspection system directly impacts the quality and consistency of data acquisition tasks, especially when operating over long distances and multiple heights. On-site experiments have been performed to evaluate the overall stability of the system, as shown in Figure 16. During this process, an inertial measurement unit (IMU) logger, mounted on the Z-axis arm, is selected as the sensing device to capture vibration and motion fluctuations along all three axes. Specific parameters of the chosen IMU sensor are presented in Table 2.

The experiment aims to record both linear acceleration and regular velocity data in real-time throughout the inspection. These data are later used for statistical analysis to evaluate the mechanical stability of the system. During the on-site experiments, the sampling frequency is set to 10 Hz, ensuring sufficient temporal resolution for detecting subtle vibrations or instabilities.

#### 2.4.2. Motion and Positioning Accuracy of the Inspection System

To precisely navigate around the rabbit house is of great importance for collecting multi-source images and environmental data. In this research, ten predesigned inspection paths are programmed into the main controller, and each route is tested individually through on-site experiments.

**Figure 17:**
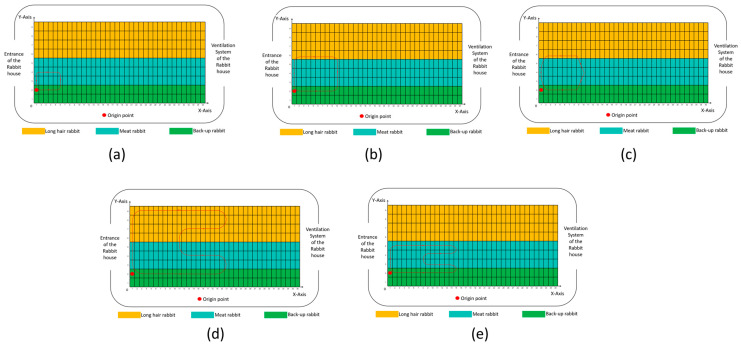
Predesigned inspection routes. (**a**–**e**) Five predesigned inspection routes selected for system validation.

During the experiment, the inspection system operates at a speed of 0.05 m/s along both the X and Y axis. Of the five selected routes shown in Figure 17, the system consistently follows the planned trajectories with high precision, indicating that the actual paths are in full agreement with the predesigned inspection routes during autonomous operation.

#### 2.4.3. Comprehensive Multi-Source Data Collection

Data completeness is essential to ensure the reliability of the inspection system. To evaluate the performance of LIVEMOS-G in this regard, we design a verification process focused on both image and environmental data streams.

For environmental data, the number of expected data records is first compared with the number of actual entries to evaluate data loss or omissions. The collected CSV files are then parsed, and each recorded value is cross-validated against measurements from high-precision reference instruments. Deviations and relative errors are calculated to determine the accuracy and consistency of the acquisition process.

For image data, all image files generated during the inspection sessions are retrieved from the specified host computer directory. Evaluation metrics include the number of all images collected, the number of each type of image, filename accuracy, and image quality. These parameters altogether serve as indicators to verify whether the imaging process meets the completeness and accuracy required for downstream analysis.

#### 2.4.4. Dead Rabbit Detection Based on LIVEMOS-G

Using the LIVEMOS-G system, multiple types of images were collected from 27 May 2024 to 21 August 2024, and an original dataset was established for the training purpose of the dead rabbit detection model. To further enrich the dataset and improve model robustness, additional images were manually collected, during which 134 deceased rabbits were identified, including 25 long-haired and 47 meat rabbits at approximately 20 days old, as well as 28 long-haired and 34 meat rabbits at approximately 55 days old. In total, 2491 sets of images were obtained, 2325 of which were selected based on image quality, 2003 sets of which contained both live and deceased rabbits, while 322 sets included only live rabbits. Each set of collected images included an RGB image (1920 × 1080 pixels), a 16-bit depth image (1080 × 768 pixels), a near-infrared image (1080 × 768 pixels), and a thermal infrared image (256 × 192 pixels). Fused images of NIR and TIR are generated using the image registration and fusion algorithms presented in Section 2.3.2. A fusion-based dead rabbit detection model under the operating scenarios of the LIVEMOS-G system was trained and further deployed for on-site experiments. For data preparation, the image annotation tool LabelImg was used to manually label the targets, marking each rabbit as alive or dead within the bounding boxes. Each dataset was randomly divided into 70% for training, 15% for validation, and 15% for testing, ensuring a balanced representation of both classes. Using these annotated images, three independent datasets were constructed to evaluate the model’s generalization and robustness under different conditions. Dataset 1 contains only dead rabbits, dataset 2 includes both dead and live rabbits in each image, and dataset 3 contains only live rabbits. For each dataset, corresponding NIR, TIR, and NIR–TIR fused images are provided. The dead rabbit detection model is then trained separately on these datasets, and a comparative analysis is conducted to evaluate its performance across different datasets and imaging modalities.

**Figure 18:**
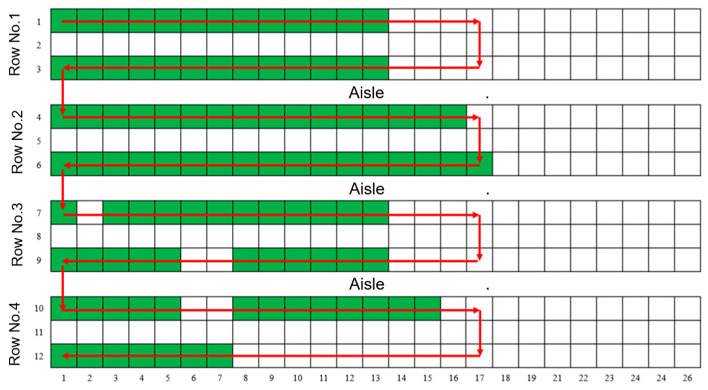
The predesigned inspection path and conditions of the cages. A green box indicates that the cage contained rabbit(s). In each row, the upper and lower rows of boxes represent the lower side-cages, while the middle row represents the upper cage.

To further evaluate the potential application of LIVEMOS-G in dead rabbit detection tasks, model testing was conducted in a commercial rabbit farm in Shandong province, China, from 5 January to 12 January 2025. During the experimental inspections, the gantry collected images for cages in each row from three various viewpoints. For the testing rabbit house served as a reserved rabbit house, only the lower cages contained rabbits. Therefore, the inspection paths did not cover upper cages, nor the parts where no rabbits were kept. The designed inspection route and conditions of the rabbit cages during the experiment are presented in Figure 18.

The pre-trained dead rabbit detection algorithm was deployed on the server to perform real-time analysis of the collected images and return detection results. Following the designed inspection process, the gantry system collected images of the 600 existing rabbits within the house. Among them, 567 sets of images contained living rabbits solely, while 61 sets included both the dead and the alive. This experiment provides data support for verifying both the performance of the algorithm and the application potential for the LIVEMOS-G system.

## 3. Results and Discussion

### 3.1. Motion and Positioning Accuracy

The mechanical stability tests were performed during 11 November 2024 to 13 November 2024, during which a total of 7317 sets of data were collected under various operating scenarios. Each set of data included the acceleration of XYZ-axes (g), the angular velocity of XYZ-axes (deg/s), and the specific angle of XYZ-axes (deg). The former parameters are chosen as indicators for steady operation. Some of the collected information are shown in Table 3.

**Figure 19:**
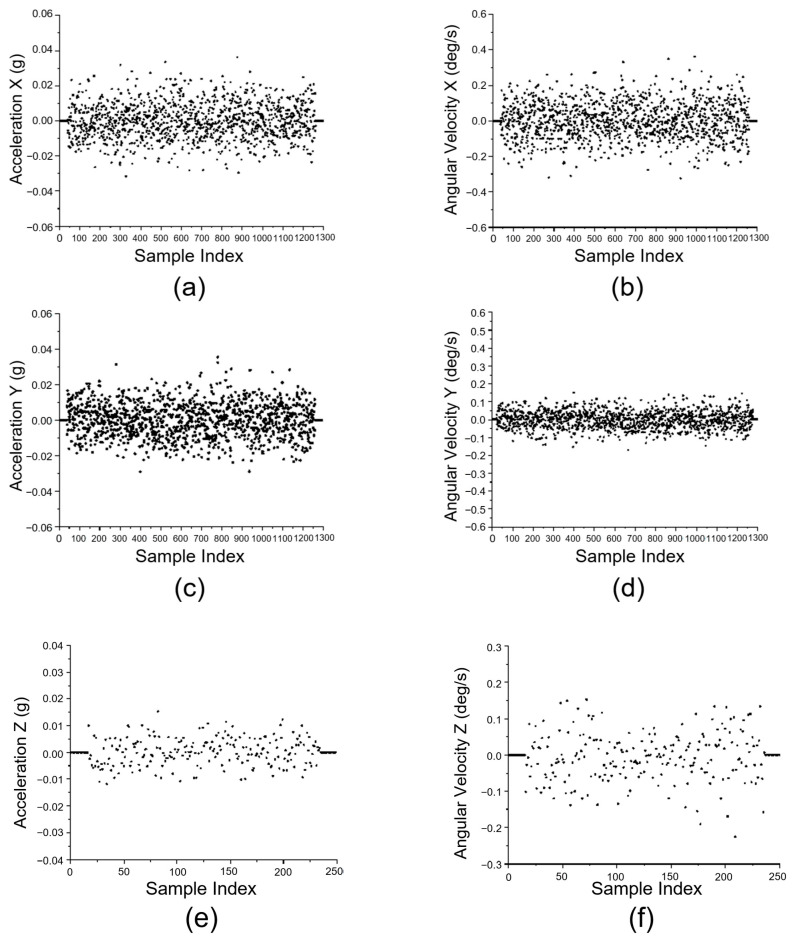
Test results from the motion stability experiment. (**a**) Acceleration along X-axis; (**b**) Angular velocity of the X-axis; (**c**) Acceleration along Y-axis; (**d**) Angular velocity of the Y-axis; (**e**) Acceleration along Z-axis; (**f**) Angular velocity of the Z-axis.

Among various validation tests, 1300 sets of data are used for XY-axes stability analysis, while 250 sets were used for Z-axis stability analysis. Data collected for motion stability confirmation are analyzed and visualized in the scatter plot, as shown in Figure 19, among which 1300 sets data regarding X-axis and Y-axis are shown in (a)–(d), and 250 sets of data regarding Z-axis are shown in (e) and (f).

Statistical analysis indicates that the inspection system demonstrates a high level of operational stability during automatic inspection. Specifically, acceleration along the X- and Y-axis remains within the range of −0.02 g to 0.02 g, while angular velocity varies between −0.2°/s and 0.2°/s along the X-axis, and −0.1°/s to 0.1°/s along the Y-axis. The Z-axis shows even smaller fluctuations, with acceleration ranging from −0.01 g to 0.01 g and angular velocity confined to −0.05°/s to 0.05°/s.

### 3.2. Environmental and Visual Data Acquisition

**Figure 20:**
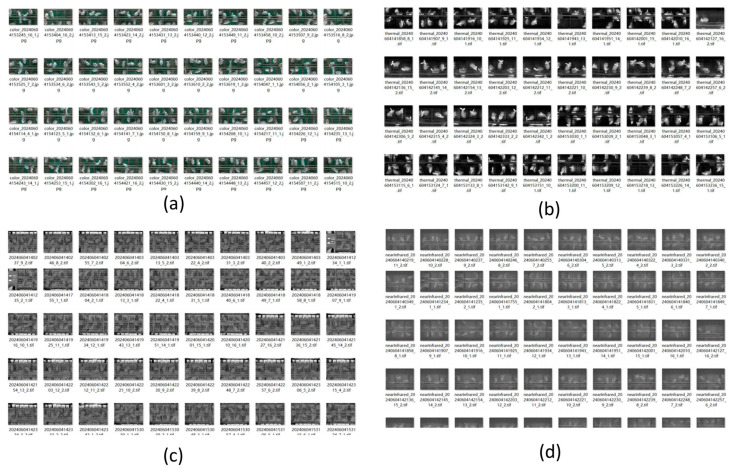
Images collected during the inspection process. (**a**) RGB images; (**b**) TIR images; (**c**) Depth images; (**d**) NIR images.

Based on the completed positioning accuracy tests of the inspection system, integrity tests are further conducted for multi-source image and environmental data acquisition. Images collected during the inspection are shown in Figure 20, showing reliable results of the multi-source imaging process.

The experiment follows the inspection route illustrated in Figure 17a, which marks ten fixed key positions, guiding the data collection process. Each inspection session captures a total of 40 images, including ten near-infrared (NIR), ten RGB, ten depth, and ten thermal infrared (TIR) images. Examination of the designated storage on the host computer confirms that all 40 images are correctly acquired during each inspection, achieving a 100% completion rate and fully meeting the stringent experimental requirements. Image naming accuracy also reaches 100%, reflecting the high operational standards and providing a solid foundation for subsequent data processing and analysis.

Meanwhile, environmental data collection involves six types of sensors measuring carbon dioxide (CO_2_), ammonia (NH_3_), atmospheric pressure, wind speed, illumination, and temperature-humidity. Partial results of the environmental data collected and stored on the host computer are presented in Table 4, including the specific numbers, as well as the time and location of these data.

**Figure 21:**
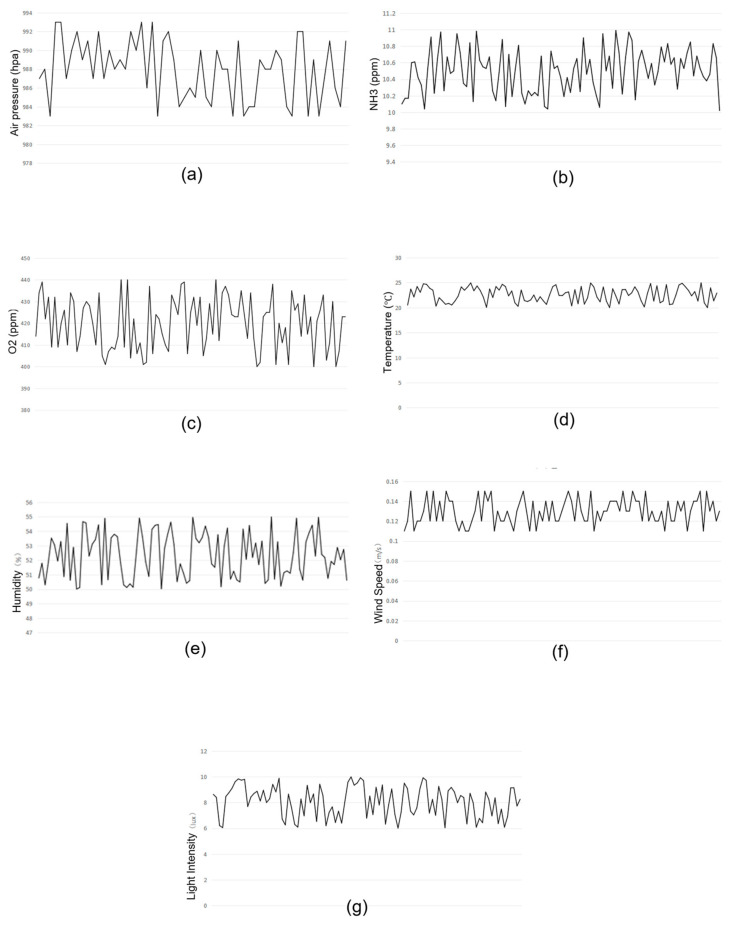
Environmental data collected during the inspection process. (**a**) Air pressure; (**b**) NH_3_; (**c**) CO_2_; (**d**) Temperature; (**e**) Humidity; (**f**) Wind speed; (**g**) Light intensity.

As shown in Figure 21, the line charts generated from these environmental data reveal variations in the environmental conditions within the rabbit house.

Rabbits are highly sensitive to environmental changes throughout courses of life. Factors such as light, temperature, humidity, ventilation, noise, harmful gases, and pathogens may directly do harm to their health and therefore affect the overall productivity. Therefore, maintaining suitable environmental conditions is essential for optimal husbandry. Typically, ammonia concentration is controlled below 20 ppm, CO_2_ levels below 3000 ppm, temperature maintained between 15 and 25 °C, humidity kept at 60–70%, as illumination intensity maintained within 50–100 lux with 12–16 h of lighting per day. Wind speed is ideally maintained between 0.1 m/s and 0.2 m/s to avoid stress caused by strong airflow, while atmospheric pressure is allowed to remain at natural levels.

According to the collected data, atmospheric pressure fluctuates between 970 and 1000 hPa. Ammonia concentration ranges from 10 to 11 ppm, mainly influenced by hygiene and animal waste within the housing. CO_2_ levels vary between 400 and 440 ppm, correlating with the rabbits’ respiration. Temperature readings fluctuate between 20.0 °C and 25.0 °C, while relative humidity remains between 50% and 55%. These variations are likely related to farming density, animal activity, and external climate conditions. Wind speed measurements range from 0.11 to 0.15 m/s, influenced by ventilation efficiency. Illumination varies between 6 and 10 lux due to various lighting conditions and cage shading.

To ensure measurement reliability of the environmental sensing module used in this study, comparative calibration experiments were conducted against reference-grade instruments. Measurements of temperature, humidity, and CO_2_ concentration were performed simultaneously at different positions and time points within the rabbit house. For each environmental condition, four repeated measurements were collected, resulting in a total of twelve data samples per parameter. A standard portable thermo-hygrometer (Testo 605i, Testo SE & Co. KGaA, Titisee-Neustadt, Germany) was used as the reference for temperature and humidity, while a commercial CO_2_ detector (Delixi Electric Co., Ltd., Leqing, China) served as the reference for CO_2_ concentration. The nominal uncertainties of these reference instruments were ±0.5 °C for temperature, ±3% RH for humidity, and ±(40 ppm + 3% of the measured value) for CO_2_, respectively.

The temperature sensor exhibited a mean measurement error of +2.2 °C, with an expanded uncertainty of ±3.1 °C under a 95% confidence level (α = 0.05). For humidity, a mean error of −1.0% RH was observed, with an expanded uncertainty of ±2.8% RH. When combined with the nominal uncertainty of the reference instrument, the total uncertainty reached ±4.1% RH. The CO_2_ sensor showed a mean error of +165 ppm, and an expanded uncertainty of ±22 ppm; considering reference uncertainty, the total combined uncertainty was ±60 ppm. These results confirm that the sensing module delivers stable and repeatable environmental measurements suitable for continuous operation in livestock housing conditions.

Analysis of real-time environmental data from additional CSV files, combined with the rabbits’ growth status, confirms that all sensor measurements fall within acceptable ranges, ensuring the high quality of the collected data, and therefore further contributing to supporting the environmental management of the rabbit house. This result indicates that the current inspection system, equipped with environmental sensors, meets multiple real-time data acquisition needs while maintaining high accuracy and reliability.

### 3.3. Possible Application in Rabbit Mortality Monitoring

As illustrated in Table 5, the proposed fusion-based dead rabbit detection model achieved excellent performance, outperforming both single source image datasets.

For Dataset 1 (containing only dead rabbits), the fused-modal model outperformed both NIR and TIR single modalities across all metrics. Precision increased from 0.955 (NIR) and 0.967 (TIR) to 0.980; Recall, which is a crucial metric for minimizing missed detections of dead rabbits, improved notably from 0.884 (NIR) and 0.952 (TIR) to 0.964; mAP@50 rose from 0.957 (NIR) and 0.986 (TIR) to 0.991, while mAP@50-95 enhanced from 0.631 (NIR) and 0.720 (TIR) to 0.737. For Dataset 2, which included rabbits both dead and alive, the fused-modal model’s overall performance (Fused (overall)) also surpassed single modalities. Precision reached 0.970, which is quite comparable to TIR (overall)’s 0.971 and higher than NIR (overall)’s 0.947; Recall achieved 0.970, surpassing NIR (overall)’s 0.916 and TIR (overall)’s 0.953; mAP@50 reached 0.990, exceeding NIR (overall)’s 0.970 and TIR (overall)’s 0.988; and mAP@50-95 reached 0.719, outperforming NIR (overall)’s 0.643 and matching TIR (overall)’s 0.712. In Dataset 3, the fused-modal model maintained competitive performance. Recall matched the high value of 0.985 (same as TIR); mAP@50 remained at 0.994 (on par with NIR and TIR); and mAP@50-95 reached 0.783, balancing performance between NIR (0.722) and TIR (0.791).

Across datasets, the fused model consistently exhibited superior or comparable performance. It should be noted that the improvements in Recall are quite more significant than the other metrics (e.g., in Dataset 1, Recall increased by 9.1% from TIR and 9.1% from NIR), which a key advantage for reliable dead rabbit detection, as higher Recall reduces the risk of failing to identify dead rabbits.

**Figure 22:**
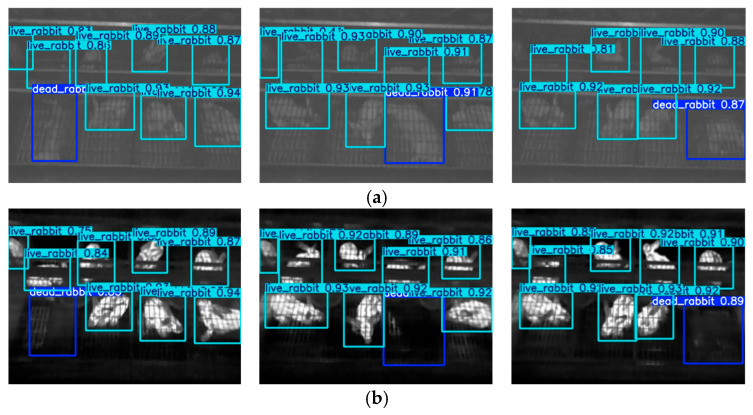

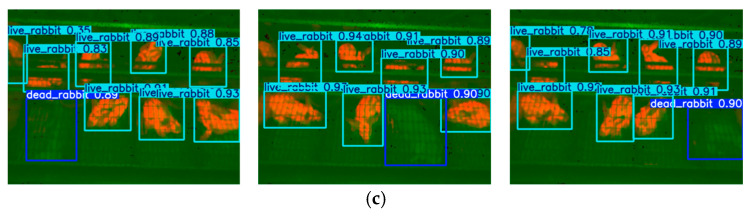
Death detection results in which all dead and live rabbits are accurately detected, based on (**a**) NIR image, (**b**) TIR image, and (**c**) fused image. A light-blue box indicate that the model detects a live rabbit while a dark-blue box indicates a detected dead rabbit.

**Figure 23:**
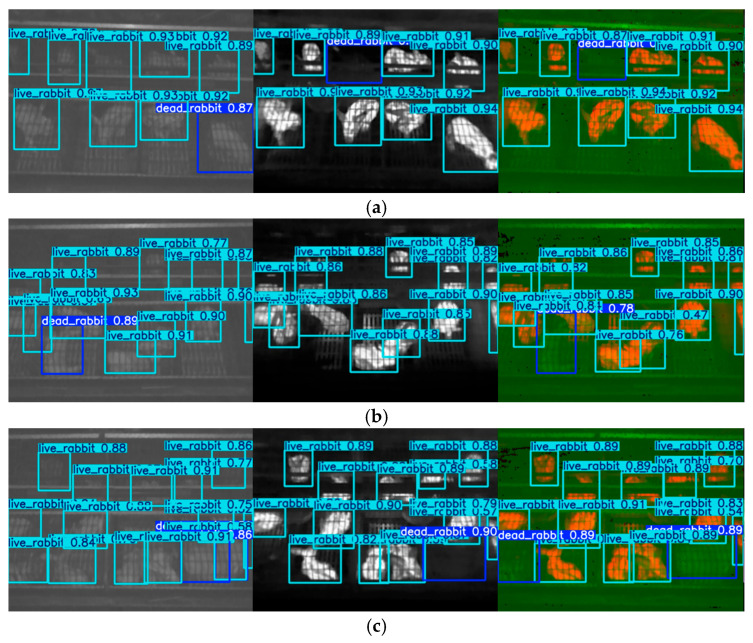
Death detection results in which fusion-based model outperformed single-source-base models. (**a**) false positive and false negative detection result based on NIR image; (**b**) false positive detection results based on TIR image; (**c**) false positive detection result based on NIR image and missed detection based on TIR image. A light-blue box indicate that the model detects a live rabbit while a dark-blue box indicates a detected dead rabbit.

Some of the detection results are shown in Figure 22 and Figure 23. The proposed method was able to accurately detect both dead and live rabbits, generating bounding boxes closely aligned with the animal contours. It also demonstrated strong robustness when handling small targets, occlusions, and variations in rabbit pose and size. This improvement is mainly attributed to the complementary properties of NIR and TIR images: NIR images provide clear structural contours even in dim environments, while TIR images supply reliable temperature information as an indicator of mortality. By fusing these modalities, the model benefits from both structural and thermal cues, resulting in enhanced robustness and improved detection accuracy under complex farming conditions.

The operational reliability of LIVEMOS-G is further validated through real-time image sharing. The operational staff are able to receive timely detection results and respond accordingly, enhancing on-site management, ensuring both the efficiency and safety of inspection tasks while minimizing the risk of false negative or false positive results.

By comparing the number of actual mortalities with the detection results, the performance of the detection network and the applicational potential of LIVEMOS-G are confirmed. In future work, we plan to further refine the detection network to improve its robustness and accuracy under diverse environmental conditions, and to explore broader application scenarios to support wider adoption across diverse farming environments.

### 3.4. Limitations and Future Works

The primary goal of the gantry-based inspection system is to enable automated inspections in large-scale rabbit farms and to collect multi-source imaging and environmental data to support farm management. This aligns with the broader concept of PLF, which aims to manage individual animals through continuous, real-time monitoring of health, welfare, production, and environmental conditions. By integrating imaging sensors, robotics, and data analytics, the proposed system contributes to the realization of PLF principles, offering a practical pathway toward intelligent and welfare-oriented livestock management. However, observations during the on-site experiments reveal several limitations:

(1) The active wheel of the LIVEMOS-G may experience slippage during inspections, resulting in ineffective trajectory correction that requires human intervention. This is mainly due to the slight slope on the right rail of the X-axis and the height differences at the rail joints, which compromise the traction and stability of the active wheels. To address this, we recommend re-evaluating and adjusting the installation of the right-side X-axis rail to improve system stability and autonomous trajectory correction capabilities.

(2) The real-time environmental data collected by the sensors are not directly integrated with the control system of the rabbit house, therefore disallowing the ventilation devices such as curtains and fans to operate autonomously under the guidance of the acquired environmental data. To enhance its potential application values, we suggest establishing a connection between the environmental sensing module and the rabbit house control cabinet in future work, allowing real-time data to guide environmental regulation. This would improve both management efficiency and environmental comfort within the housing unit.

## 4. Conclusions

This study introduces LIVEMOS-G, a gantry-type multi-source inspection platform designed to meet the evolving demands of large-scale rabbit farming. The system combines a three-axis motion structure with multi-source imaging modules and environmental sensing modules, enabling fully automated, high-precision inspections across complex cage environments. Unlike traditional manual inspections or ground-based mobile platforms, LIVEMOS-G provides stable and adjustable viewpoints for sensors from above the cages, significantly improving data coverage, hardware adaptability, and the overall data quality.

Mechanical validation experiments confirmed the structural stability of the system during operation, with minimal vibration and high motion accuracy. The inspection process consistently achieved complete and accurate collection of multi-source image and environmental data. As for potential applications, the system’s integration with a fusion-based dead rabbit detection algorithm enabled real-time mortality monitoring with high accuracy and robustness. Environmental sensing module is able to collect six environmental parameters essential for rabbit welfare, offering critical data for management decisions. As the same cage layouts are widely applied in rabbit houses around Europe as well as east Asia, and that the rabbit breeds involved in the experiments, namely Angora long-haired rabbit and Hyla meat rabbit, are commonly raised in these places, the proposed system shows promising generalizability.

Despite its promising performance, the system currently faces limitations in motion correction under specific physical conditions, and the environmental data is not yet connected to autonomous environmental control systems within the farm.

Future work will focus on improving the mechanical robustness of the gantry structure and further optimizing the detection algorithms under varying environmental conditions. In addition, the integration of smart environmental control devices, for example, linking real-time environmental data with the control system of the ventilation systems, will be explored to enable closed-loop environmental management. In addition, we also aim to realize cross-species death detection, in which a single detection model is able to detect multiple dead species and can be applied to various livestock or poultry farms, so as to further improve the general application potential of the LIVEMOS-G system. Moreover, illness detection is another prospect. If the unwell animals can be detected pre-mortality, and are isolated to be specially treated, the risk of pollution and cross-infection can be minimized. In all, we plan to achieve the incorporation of predictive analytics into animal health and welfare assessment in the future, further working towards the fully intelligent and sustainable livestock farming.

Future work aims to enhance mechanical robustness, and further optimize image analysis algorithms under varying environmental conditions.

The development of LIVEMOS-G represents an important step toward intelligent, welfare-oriented animal farming. By reducing reliance on manual labor, enhancing monitoring precision, and enabling timely intervention, such systems address key challenges in modern livestock production. In the context of rising global food demands and heightened awareness of animal welfare, novel monitoring systems like LIVEMOS-G contribute not only to improving farm-level efficiency, but also to ensuring food security, safeguarding animal health, and promoting sustainable agricultural practices. As PLF continues to evolve, gantry-based intelligent inspection platforms offer a promising foundation for the next generation of fully automated, and data-driven farming systems.

## Figures and Tables

**Table 1:** Details regarding the environmental sensors chosen.

Communication Protocol	Sensor	Unit	Measurement Range	Accuracy
Modicon Bus (Modbus)	Air pressure sensor	hPa	26–126 kPa	±20 Pa
	CO_2_ sensor	PPM	0–2000 ppm	±(40 ppm ± 3%F·S)
	NH_3_ sensor	PPM	0–100 ppm	±(3 ppm + 3%F·S)
	Wind speed sensor	M/S	0–1 m/s	3%
Inter-integrated circuit (IIC)	Light sensor	Lux	0–8800 lux	188 ulux
	Temperature humidity sensor	°C, %	−40–125 °C, 0–100%RH	±0.3 °C, ±3%RH

**Table 2:** Specific parameters of the IMU sensor logger.

Specification	Parameter
Model	WT901SDCL-BT50
Communication Method	Type-C USB serial communication, Bluetooth communication
Measurement Dimensions	Three-axis (acceleration, gyroscope, angle, magnetic field), quaternion
Angular Accuracy	X and Y axis: 0.2°, Z axis: 1° (under magnetic-field-free conditions and after calibration)
Sampling Frequency	0.1–200 Hz

**Table 3:** Samples of the collected data.

Time	Acceleration X (g)	Acceleration Y (g)	Acceleration Z (g)	Angular Velocity X (deg/s)	Angular Velocity Y (deg/s)	Angular Velocity Z (deg/s)
15:42:48.948	0.021	0.005	0.014	0	0.027	−0.022
15:42:49.038	−0.012	0.005	0.017	0	0.016	−0.022
15:42:49.158	0.006	0.011	0.048	0.193	0.065	−0.048
15:42:49.217	0.011	0.006	0.017	−0.122	−0.097	0
15:42:49.367	0.016	0.004	0.022	−0.205	−0.115	0
15:42:49.427	0.014	0.005	0.012	−0.183	−0.166	0.061
15:42:49.547	0.018	0.004	0.018	−0.183	−0.104	0.066
15:42:49.638	0.014	0.005	0.013	−0.122	−0.088	0.044
15:42:49.758	0.014	0.003	0.02	−0.061	0.071	0.044
15:42:49.848	0.017	0.003	0.013	0.061	0.068	0.021

**Table 4:** Samples of the collected environmental data.

Time	Wind Speed (m/s)	Air Pressure (hPa)	NH_3_ (ppm)	CO_2_ (ppm)	Temperature (°C)	Humidity (%)	Light Intensity (lux)	Sample Location
0604-10:53:45	0.13	990	10.68	410	20.87	51.43	6.44	1-1
0604-10:53:54	0.15	983	10.73	410	20.84	51.42	6.51	2-1
0604-10:54:03	0.13	987	10.08	409	20.85	51.44	6.45	3-1
0604-10:54:12	0.13	987	10.86	409	20.88	51.46	6.71	4-1
0604-10:54:23	0.13	985	10.5	410	20.85	51.51	6.28	5-1
0604-10:54:30	0.14	985	10.45	409	20.83	51.49	6.24	6-1
0604-10:54:39	0.15	988	10.13	413	20.85	51.52	6.34	6-2
0604-10:54:47	0.13	983	10.01	414	20.84	51.45	5.92	5-2
0604-10:54:57	0.13	985	10.02	413	20.84	51.49	5.44	4-2
0604-10:55:05	0.14	983	10.38	413	20.83	51.43	6.24	3-2

**Table 5:** Comparison of the training results of the dead rabbit detection model on different image modalities. Underlined results indicate best performance among the same dataset.

Dataset		Performance			
		Precision	Recall	mAP@50	mAP@50-95
Dataset 1	NIR	0.955	0.884	0.957	0.631
	TIR	0.967	0.952	0.986	0.720
	Fused	0.980	0.964	0.991	0.737
Dataset 2	NIR (dead)	0.932	0.882	0.957	0.635
	NIR (live)	0.961	0.950	0.983	0.651
	NIR (overall)	0.947	0.916	0.970	0.643
	TIR (dead)	0.971	0.943	0.985	0.708
	TIR (live)	0.971	0.962	0.990	0.717
	TIR (overall)	0.971	0.953	0.988	0.712
	Fused (dead)	0.977	0.967	0.991	0.720
	Fused (live)	0.963	0.972	0.990	0.717
	Fused (overall)	0.97 0	0.97 0	0.99 0	0.719
Dataset 3	NIR	0.977	0.982	0.994	0.722
	TIR	0.989	0.985	0.994	0.791
	Fused	0.977	0.985	0.994	0.783

## Data Availability

The data are not publicly available due to privacy reasons.
